# Influence of remnant lipoprotein particle cholesterol on non-target lesions progression in patients undergoing percutaneous coronary intervention

**DOI:** 10.3389/fcvm.2024.1471479

**Published:** 2024-12-10

**Authors:** Jing Liu, Tian-Qi Teng, Zheng Li, Feng-Wang Hu, Wei-Wei Sha, Chang-Xian Shen, Yong Xia, Yao-Jun Zhang, Li Liang

**Affiliations:** ^1^ Department of Neurology, Xuzhou New Health Geriatric Hospital, Xuzhou, Jiangsu, China; ^2^ Department of Cardiology, The Affiliated Hospital of Qingdao University, Qingdao, Shandong, China; ^3^ Department of Cardiology, Xuzhou New Health Geriatric Hospital, Xuzhou, Jiangsu, China; ^4^ Department of Cardiology, Institute of Cardiovascular Diseases, Xuzhou Medical University, Xuzhou, China

**Keywords:** atherosclerosis, percutaneous coronary intervention, remnant lipoprotein particle cholesterol, triglyceride-rich lipoproteins, non-target lesion

## Abstract

**Background:**

Low-Density Lipoprotein Cholesterol (LDL-C) is the primary lipid therapy target for coronary artery disease (CAD) patients after percutaneous coronary intervention (PCI). However, progression of coronary atherosclerosis occurs even LDL-C controlled well, some potentially important factors have been overlooked.

**Objective:**

This study aims to elucidate the relationship between remnant lipoprotein particle cholesterol (RLP-C) and the progression of non-target lesions (NTLs) in patients with well-controlled lipid levels after PCI.

**Methods:**

This retrospective study included 769 CAD patients who underwent PCI and followed up angiography within 6–24 months thereafter. Employing Multivariate Cox regression analysis, we assessed the correlation between RLP-C and NTLs progression. Based on the receiver operating characteristic curve analysis, we identified the optimal cutoff point for RLP-C, following which the patients were divided into two groups. Propensity score matching balanced confounding factors between groups, and Log-rank tests compared Kaplan–Meier curves for overall follow-up to assess NTLs progression.

**Results:**

Multivariate Cox analysis revealed an independent association between RLP-C and NTLs progression when LDL-C was well-controlled. Additionally, the RLP-C level of 0.555 mmol/L was determined to be the best value for predicting NTLs progression. Following propensity score matching, Kaplan–Meier curves illustrated a significantly higher cumulative rate of NTLs progression in patients with RLP-C levels ≥0.555 mmol/L compared to the others (Log-rank *P* = 0.002). Elevated RLP-C levels were associated with high triglyceride concentrations, diabetes mellitus, and increased risk of revascularization.

**Conclusions:**

This study illustrated the atherogenic impact of RLP-C in CAD patients. High RLP-C levels increased the risk of revascularization.

## Introduction

Low-Density Lipoprotein Cholesterol (LDL-C) has received sufficient attention from clinicians in patients with coronary artery disease (CAD) undergoing percutaneous coronary intervention (PCI). However, cardiovascular events persist despite LDL-C has been managed to recommended levels and other common risk factors have been addressed (1). The ESC/EAS Guidelines for the management of dyslipidemias (2019) have established stricter criteria for LDL-C in patients at very high cardiovascular risk, while also emphasizing the importance of other lipid components (2). Genetic and epidemiological evidence indicates that elevated levels of Remnant Lipoprotein Particle Cholesterol (RLP-C) are a significant factor contributing to the residual risk of atherosclerotic cardiovascular disease (ASCVD) (3–5).

RLP-C, the cholesterol content carried by Triglyceride-rich lipoproteins (TRL), includes very low-density lipoprotein and intermediate-density lipoprotein in the fasting state, as well as chylomicrons in the non-fasting state. Elevated TRL levels transport cholesterol that permeates the arterial wall, leading to foam cell formation, atherosclerosis, and both localized and systemic inflammation (6). Recent research from the UK Biobank found that TRL demonstrates substantially greater atherogenicity per-particle than LDL (3). Almost 50% of recurrent cardiac ischemic events stemmed from the progression of NTLs following successful PCI (7). However, research on the risk of NTLs progression after PCI is not extensive, especially in patients with LDL-C levels well controlled.

This paper aims to establish a novel target for the prevention and treatment of such patients by examining the correlation between RPL-C and the advancement of non-target lesions, scrutinizing the clinical attributes of individuals with elevated RLP-C levels, and laying a foundation for post-PCI patient management.

## Materials and methods

### Study patients

**Figure 1:**
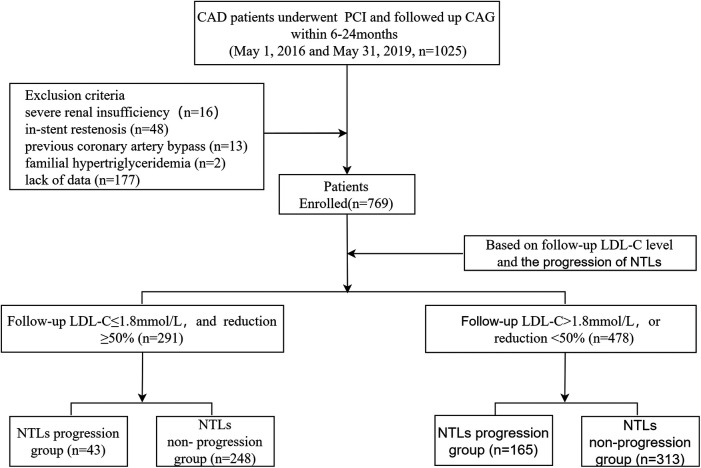
Flow chart of patient enrollment. CAD, coronary artery disease; PCI, percutaneous coronary intervention; CAG, coronary angiography; LDL-C, low density lipid-cholesterol; NTLs, non-target lesions.

We reviewed patients diagnosed with CAD who underwent successful PCI between May 1, 2016 and May 31, 2019, and followed up coronary angiography within 6–24 months. Data from 1,025 participants were initially gathered. Study exclusion criteria included severe renal insufficiency with estimated glomerular filtration rate ≤30 ml/min/1.73 m^2^ (*n* = 16); in-stent restenosis (*n* = 48); previous coronary artery bypass (*n* = 13); familial hypertriglyceridemia (*n* = 2); and lack of data (*n* = 177). A total of 769 patients meeting the inclusion and exclusion criteria were included in the analysis for this study (Figure 1). The study protocol complied with the Declaration of Helsinki and was approved by the Local Research Ethics Committee (Decision Date: 2022-12-22; Decision Numbers: IEC-C-008-A07-V1.0 and 2022-02-053-K01); all the enrolled patients gave written informed consent.

The optimal cut-off value of RLP-C was determined through receiver operating characteristic (ROC) curve analysis. Subsequently, patients were categorized into two groups: a low RLP-C group (*n* = 124) and a high RLP-C group (*n* = 166). Propensity score matching analysis was conducted between the two groups at a ratio of 1:2, incorporating baseline data such as diabetes mellitus, smoking history, presence of multiple vessel disease, High density lipoprotein cholesterol (HDL-C) levels, TG levels, and systolic pressure. Eventually, 83 cases in the high RLP-C group and 106 cases in the low RLP-C group were matched well. Then, Log-rank tests were employed to compare Kaplan–Meier curves for overall follow-up, evaluating the progression of NTLs between the two groups.

### Data collection

Gather clinical data on patients, encompassing gender, age, smoking, alcohol, past medical history. Record vital signs upon admission, including blood pressure, heart rate, height, weight, etc. Body mass index (BMI) was calculated by dividing weight in kilograms by the square of the height in meters. Coronary angiography analysis and QCA were performed by two experienced researchers.

Standardized procedures are followed for routine blood and biochemical tests, which are conducted in the fasting state. TG were assessed using the enzymatic colorimetric GPO-PAP method, TC was determined using the enzymatic-cholesterol oxidase peroxidase method, and both LDL-C and HDL-C were measured through direct homogeneous assay. RLP-C levels were derived by subtracting the sum of HDL-C and LDL-C from TC, following a standard lipid curve (8).

### Quantitative coronary angiography

The degree of coronary artery stenosis is determined through QCA analysis, which is conducted by skilled technicians blinded to patient identity and clinical profile. The QCA software was utilized to assess all three major untreated coronary vessels, covering all side branches with a reference vessel diameter greater than 1.5 mm. This evaluation was conducted using angiographic views that were comparable for the NTL segments, between the baseline and follow-up periods. Measured variables included reference vessel diameter, minimal luminal diameter, and diameter stenosis.

### Diagnostic criteria

NTLs: This coronary vascular lesion was not associated with ischemic symptoms or functional ischemia test results at baseline CAG. Coronary plaque progression: (1) Lesions with baseline diameter stenosis exceeding 50% progressed by more than 10%; (2) Lesions with initial diameter stenosis below 50%, including normal segments, progressed to over 30%; (3) Any non-target lesions progressed to occlusion. Blood lipid goals: LDL-C levels below 1.8 mmol/L with a reduction of more than 50% from baseline. Multivessel disease: two or more vessels with ≥50% diameter stenosis (9).

### Statistical analysis

Continuous variables were analyzed using two-sided *t*-tests or Mann-Whitney U tests, and described by mean ± standard deviation (SD) or medians (interquartile ranges, IQR). Categorical variables were analyzed using chi-square or Fisher's exact test and presented as counts (percentages). Covariates with a univariate analysis *P* value <0.1 or clinically relevant risk factors were incorporated into the Cox multivariate regression model to determine the correlation between RLP-C and non-target lesion progression. In order to better reflect the impact of the change of independent variables on the outcome, continuous independent variables were evenly stratified into four quartiles, with the initial quartile (Q1) as the reference.

ROC curve analysis was used to evaluate the ability of RLP-C to predict NTLs progression. Patients were then categorized into high and low RLP-C groups based on the cutoff value. Propensity score matching was used to minimize between-group discrepancies. A standardized difference of less than 0.1 indicated satisfactory covariate balance. Following several matching attempts, the 1:2 nearest neighbor matching method with a caliper value of 0.2 achieved optimal balance between the groups. Log-rank tests were employed to assess Kaplan-Meier curves over the entire follow-up period, examining the progression of NTLs between the two groups.

To assess the association between RLP-C and other lipid parameters, either Pearson's correlation analysis or Spearman's correlation analysis was employed.

Statistical analyses were conducted using SPSS software for Windows (version 22.0, SPSS Inc., Chicago, Illinois, USA). A two-sided probability value of less than 0.05 was deemed statistically significant in all analyses.

## Results

### Characteristics of the study subjects

The study enrolled a total of 769 patients, with 291 (37.84%) meeting the lipid goals, including 43 (14.78%) in the NTLs progression group. Additionally 478 (62.16%) failed to meet the lipid goals, including 165 (34.52%) in the NTLs progression group (Figure 1).

The average follow-up duration was 13.08 ± 5.2 months. Among patients meeting the lipid goals, the progression group had a shorter follow-up duration (13.34 ± 5.27 vs. 11.54 ± 4.57; *P* = 0.035), a higher rate of smoking (42.3% vs. 60.5%; *P* = 0.027) and history of diabetes mellitus (23.0% vs. 44.19%, *P* = 0.003) and MVD (34.7% vs. 58.1%, *P* = 0.003) (Table 1).

**Table 1:** Clinical and angiographic characteristics of patients met lipid goals.

Baseline Variables	Total (291)	Non progression group (248)	Progression group (43)	*t/z/χ* ^2^	*P*
follow-up period (months)	13.08 ± 5.2	13.34 ± 5.27	11.54 ± 4.57	2.115	0.035*
age (years)	65.21 ± 11.34	65.14 ± 11.72	65.63 ± 9.11	−0.259	0.796
male, *n* (%)	199 (68.4)	168 (67.5)	31 (72.1)	0.321	0.571
smoking, *n* (%)	131 (45)	105 (42.3)	26 (60.5)	4.865	0.027*
history of hypertension, *n* (%)	176 (60.5)	148 (59.7)	28 (65.1)	0.454	0.501
history of DM, *n* (%)	74 (25.4)	57 (23.0)	17 (44.19)	8.537	0.003**
BMI (Kg/m^2^)	24.62 ± 2.91	24.59 ± 2.85	24.78 ± 3.30	−0.407	0.684
SBP (mmHg)	133.41 ± 15.77	133.01 ± 16.05	135.28 ± 14.10	−0.842	0.401
DBP (mmHg)	77.57 ± 10.97	77.05 ± 10.68	80.56 ± 12.18	−1.944	0.053
heart rate (beat/min)	71.62 ± 9.79	71.88 ± 9.64	70.07 ± 10.61	1.122	0.263
SCr (umol/L)	67.38 ± 13.81	66.87 ± 14.10	70.33 ± 11.70	−1.520	0.130
BUN (mmol/L)	5.55 ± 1.83	5.54 ± 1.76	5.61 ± 2.22	−0.231	0.817
UA (umol/L)	295.25 ± 90	291.75 ± 91.34	315.42 ± 79.84	−1.596	0.112
FIB (g/L)	3.1 ± 0.8	3.09 ± 0.79	3.19 ± 0.87	−0.819	0.413
NLR	2.22 (1.73, 2.85)	2.24 (1.73, 2.85)	2.22 (1.73, 2.97)	−0.481	0.631
DAPT, *n* (%)	285 (97.9)	243 (98.0)	42 (97.7)	0.017	0.895
statin, *n* (%)	286 (98.3)	244 (98.4)	42 (97.7)	1.657	0.196
MVD, *n* (%)	111 (38.1)	86 (34.7)	25 (58.1)	8.550	0.003**

DM, diabetes mellitus; BMI, body mass index; SBP, systolic blood pressure; DBP, diastolic blood pressure; SCr, serum creatinine; BUN, bun urea nitrogen; UA, uremic acid; FIB, fibrinogen; NLR, neutrophil to lymphocyte ratio; DAPT, dual anti-platelet therapy; MVD, multivessel disease.

* *P* value < 0.05.

** *P* value < 0.01.

Compared to the non-progression group, patients with well-controlled LDL-C who still experienced NTLs progression exhibited significantly higher baseline RLP-C levels (0.70 ± 0.45 vs. 0.86 ± 0.41; *P* = 0.035) and follow-up RLP-C levels (0.54 ± 0.27 vs. 0.73 ± 0.29; *P* < 0.001), as well as higher TG levels (1.36 ± 0.63 vs. 1.71 ± 0.62; *P* = 0.001). Additionally, the progression group had significantly lower follow-up HDL-C levels (1.29 ± 0.32 vs. 1.15 ± 0.27; *P* = 0.007). No significant differences were observed in TC and LDL-C levels (*P* > 0.05) (Table 2).

**Table 2:** Lipid at baseline and follow-up.

Variables (mmol/L)	Non progression group (248)	Progression group (43)	*t/z/χ* ^2^	*P*
TC-BL	4.61 ± 0.76	4.66 ± 0.80	−0.417	0.677
TC-FU	3.34 ± 0.47	3.37 ± 0.36	−0.570	0.570
TCΔ	1.27 ± 0.66	1.29 ± 0.86	−0.127	0.880
HDL-C-BL	1.29 ± 0.22	1.23 ± 0.28	1.618	0.107
HDL-C-FU	1.29 ± 0.32	1.15 ± 0.27	2.700	0.007**
HDL-CΔ	−0.02 ± 0.38	0.076 ± 0.39	−1.230	0.218
LDL-C-BL	2.61 ± 0.61	2.57 ± 0.57	0.407	0.685
LDL-C-FU	1.50 ± 0.24	1.49 ± 0.20	0.321	0.749
LDL-CΔ	1.101 ± 0.59	1.08 ± 0.55	0.290	0.772
TG-BL	1.68 (1.38, 2.04)	1.68 (1.23, 2.43)	−0.100	0.992
TG-FU	1.36 ± 0.63	1.71 ± 0.62	−3.414	0.001**
TGΔ	0.41 ± 0.80	0.20 ± 1.18	1.097	0.278
RLP-C-BL	0.70 ± 0.45	0.86 ± 0.41	−2.123	0.035*
RLP-C-FU	0.54 ± 0.27	0.73 ± 0.29	−4.177	<0.001***
RLP-CΔ	0.16 ± 0.48	0.13 ± 0.51	0.415	0.678

BL, baseline; FU, follow-up; Δ, difference value.

* *P* value < 0.05.

** *P* value < 0.01.

*** *P* value < 0.001.

### Cox regression analysis and ROC curve

**Figure 2:**
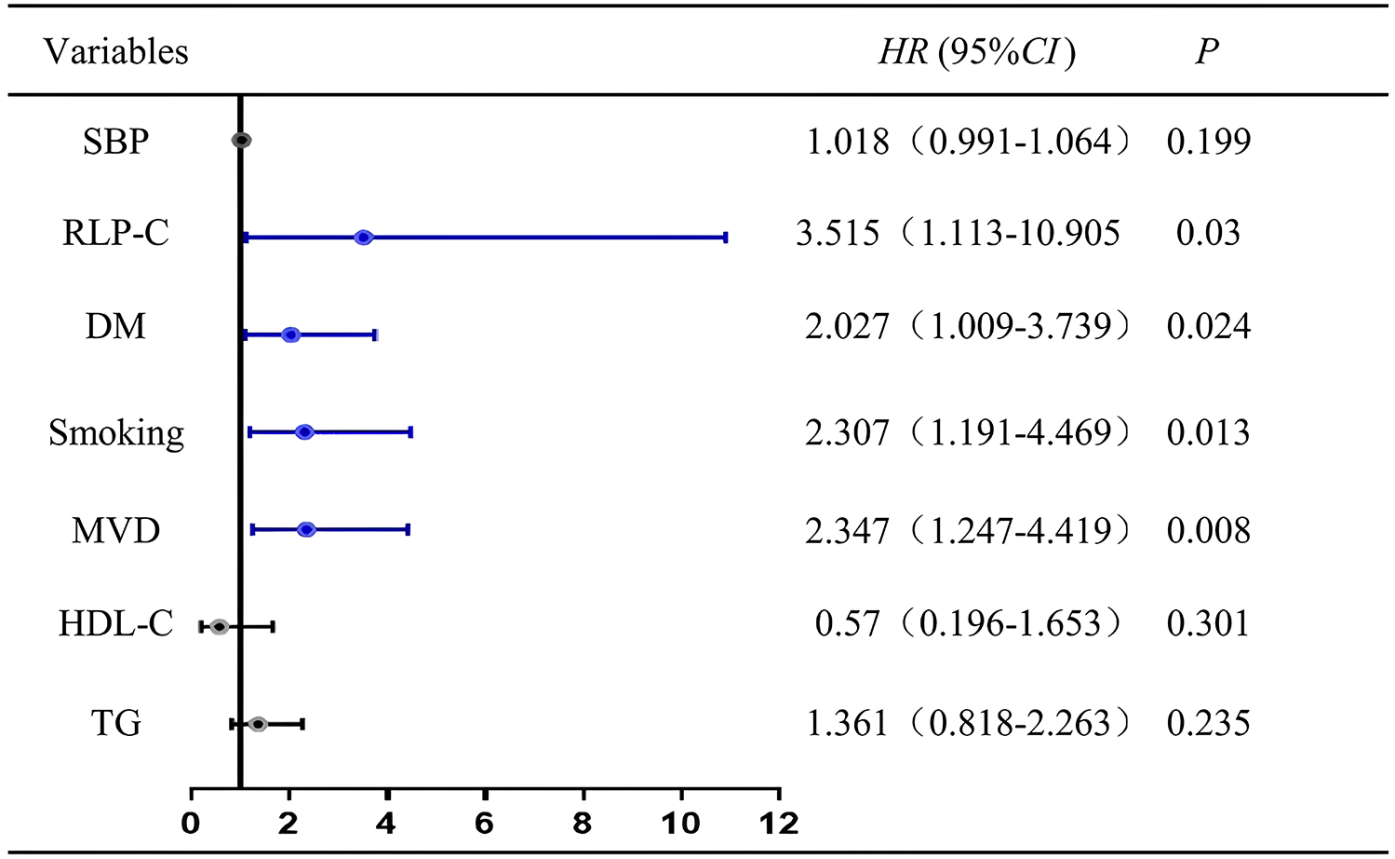
Cox regression analysis for the presence of non-target lesion progression. SBP, systolic pressure; RLP-C, remnant lipoprotein particle cholesterol; DM, diabetes mellitus; HDL-C, high density lipoprotein cholesterol; MVD, multivessel disease.

To ascertain the contribution of baseline RLP-C to NTLs progression under well-controlled LDL-C conditions, we incorporated risk factors with *P* < 0.1 or factors potentially associated with NTLs progression in clinical settings into our regression analysis, including systolic blood pressure (SBP), DM, MVD, HDL-C, and TG. After accounting for other confounding factors, the analysis showed that RLP-C, diabetes, smoking, and multi-vessel disease independently increased the risk of NTLs progression. Elevated RLP-C, as a residual lipid risk factor, significantly contributed to non-target lesion progression (HR = 3.515, 95% CI: 1.133–10.905, *P* = 0.03) (Figure 2).

**Figure 3:**
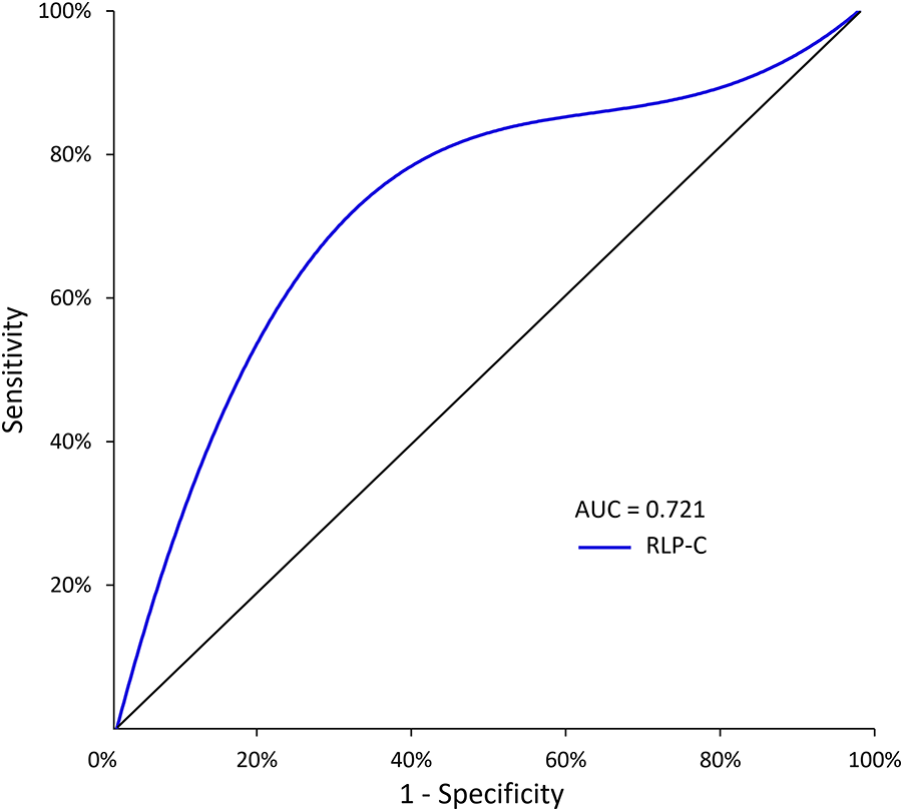
The predictive values of RLP-C level for predicting the risk of NTLs progression. RLP-C, Remnant lipoprotein particle cholesterol.

ROC curve analysis revealed an AUC of 0.721 (95% CI = 0.635–0.807, *P* < 0.001), indicating the strong predictive capability of RLP-C for NTLs progression risk in LDL well-controlled patients post-PCI. The baseline RLP-C level of 0.555 mmol/L was determined as the optimal cutoff point for predicting progression risk, with a sensitivity of 81.4% and specificity of 63.7% (Figure 3).

### Propensity score matching, clinical characteristics and Kaplan-Meier curves

**Figure 4:**
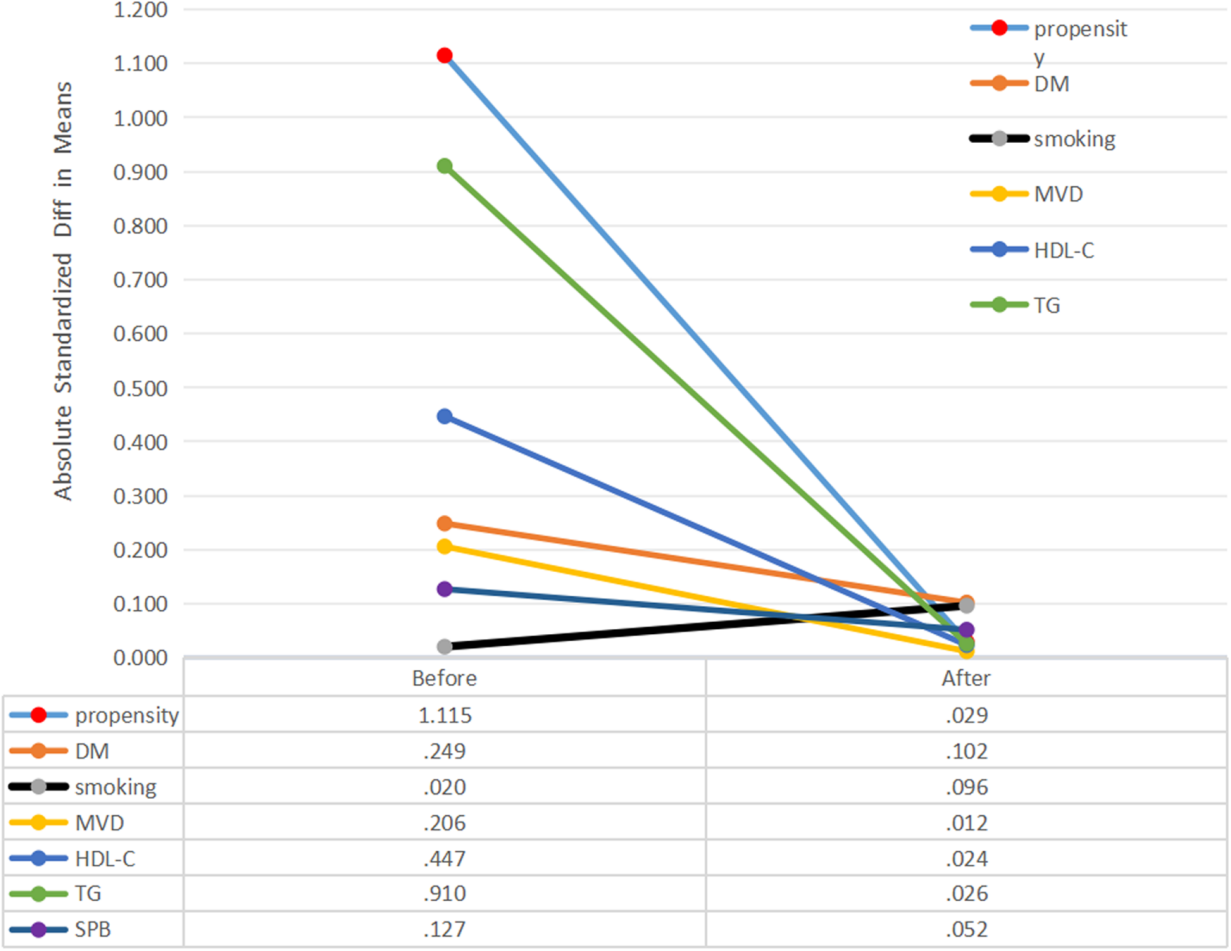
Absolute standardized differences in unweighted and propensity score-weighted data sensitivity analyses. DM, diabetes mellitus; MVD, Multivessel disease; HDL-C, high density lipoprotein cholesterol; TG, triglyceride; SBP, systolic pressure.

To further adjust for potential risk factors that could cause imbalance between the high (*n* = 124) and low (*n* = 167) RLP-C groups, Propensity Score Matching (PSM) was applied. After the PSM analysis, unmatched cases were excluded, and 83 participants were reassigned to the high RLP-C group, while 106 participants were assigned to the low RLP-C group. Notably, the balance effect of the covariates after applying PSM was deemed satisfactory (Figure 4).

**Figure 5:**
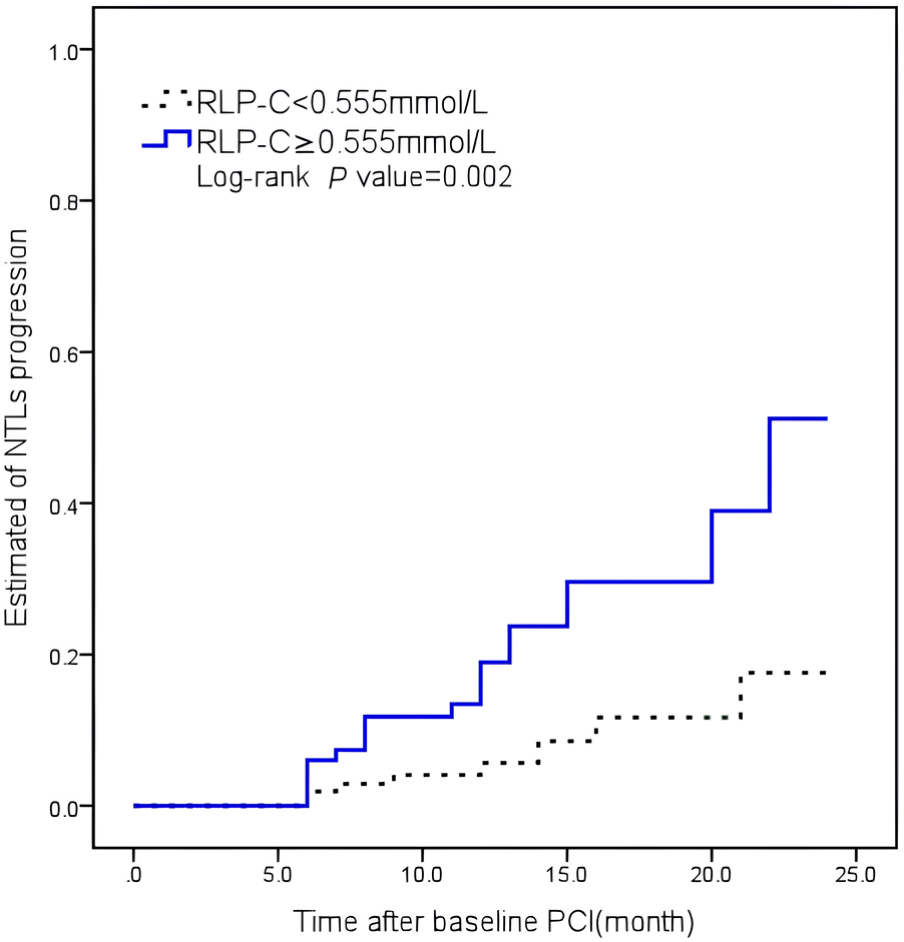
Kaplan–Meier curves illustrating the cumulative rate of non-target lesion progression. RLP-C, remnant lipoprotein particle cholesterol.

No notable variances were detected between the low and high RLP-C groups regarding DM history, smoking, multi-vessel disease, HDL-C, TG, or SBP after propensity score matching. Kaplan–Meier curves illustrated a significantly higher cumulative rate of NTLs progression in patients with RLP-C levels ≥0.555 mmol/L compared to the others (Log-rank *P* = 0.002) (Figure 5).

### Clinical characteristics of patients with high RLP-C

All participants with better LDL-C control were divided into 2 groups based on RLP-C cutoff values, regardless of whether NTL progression occurred. In patients with high RLP-C, there was a significantly higher proportion of diabetes (32.8% vs. 21.1%, *P* = 0.024), a slightly higher proportion of multi-vessel disease (44.0% vs. 33.7%, *P* = 0.074) and a shorter average follow-up duration (13.34 ± 5.27 vs. 11.54 ± 4.57, *P* = 0.035) compared to the low RLP-C group. No statistically significant differences were observed in terms of gender, age, BMI, hypertension, smoking.

**Figure 6:**
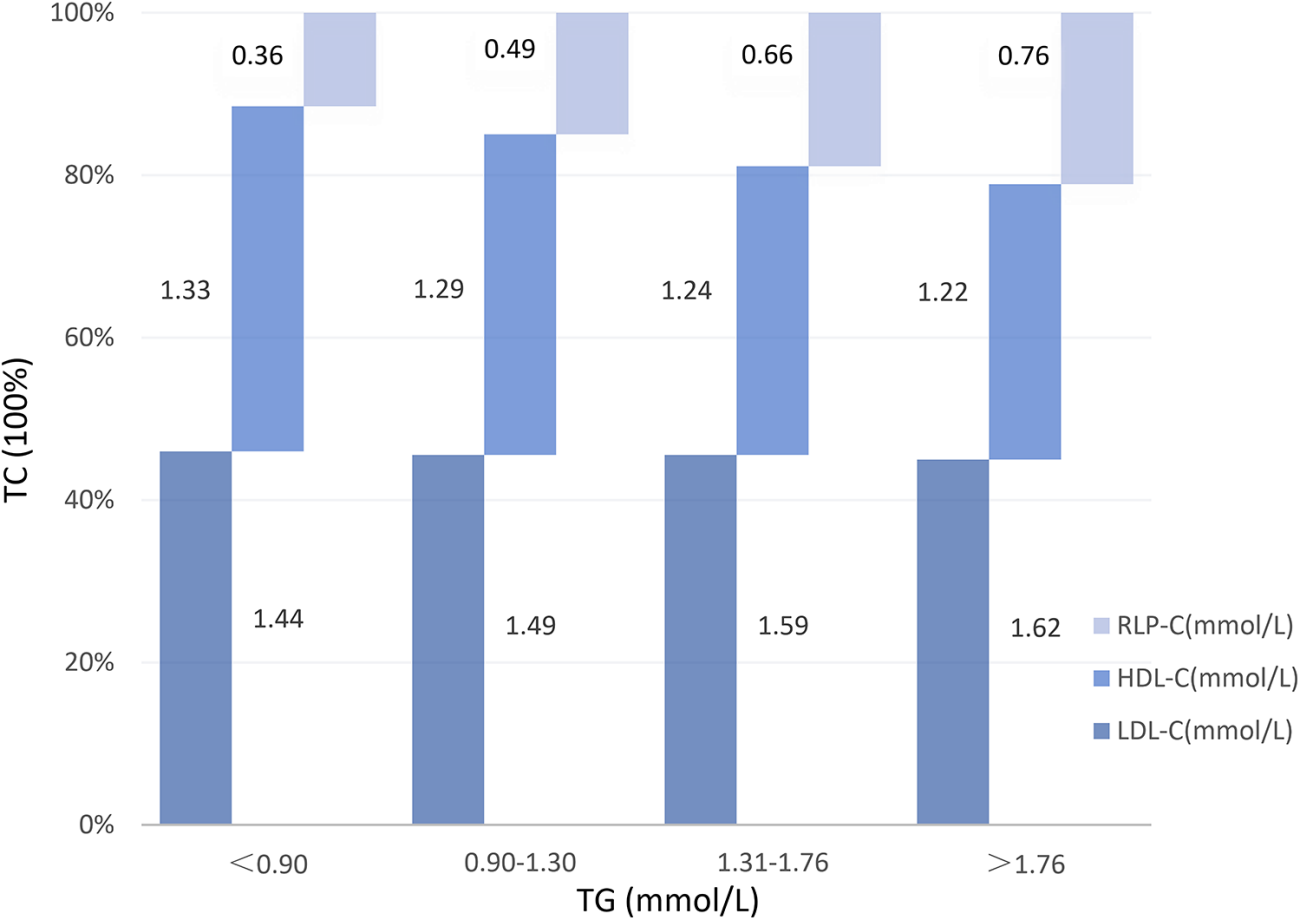
RLP-C and its proportion in TC at different TG levels in well-controlled LDL-C individuals. RLP-C, remnant lipoprotein particle cholesterol; HDL-C, high density lipoprotein cholesterol; LDL-C, low density lipoprotein cholesterol.

At baseline, no notable differences were evident in vessel lesion diameter, stenosis rate, or location between the two groups. However, during follow-up, individuals with elevated RLP-C levels exhibited markedly reduced lesion diameters (1.46 ± 0.78 vs. 1.70 ± 0.80, *P* = 0.01) and an elevated risk of revascularization (16.9% vs. 8.4%, *P* = 0.028). The incidence of myocardial infarction remained low in both groups (Table 3). Correlation analysis indicated a positive correlation between RLP-C and TG as well as TC, with the strongest correlation observed with TG (*r* = 0.583, *P* < 0.01 at follow-up; *r* = 0.551, *P* < 0.01 at baseline). RLP-C exhibited an inverse relationship with HDL-C and demonstrated no significant correlation with LDL-C (Table 4). Upon categorizing patients into four groups based on quartiles of TG levels, an increase in both RLP-C levels and its proportion in TC was observed with higher TG levels (Figure 6).

**Table 3:** Clinical characteristics of patients with high RLP-C.

Variables	High RLP-C (*n* = 124)	Low RLP-C (*n* = 167)	*t/z/χ* ^2^	*P*
Male, *n* (%)	89 (71.2)	110 (66.3)	0.803	0.445
Age (years)	65.13 ± 10.14	65.28 ± 12.23	0.114	0.910
BMI (Kg/m^2^)	24.50 ± 2.98	24.70 ± 2.87	0.561	0.575
History of DM, *n* (%)	41 (32.8)	35 (21.1)	5.072	0.024*
History of hypertension, *n* (%)	81 (64.8)	95 (57.2)	1.710	0.191
MVD, *n* (%)	55 (44.0)	56 (33.7)	3.184	0.074
Smoking, *n* (%)	57 (45.6)	74 (44.6)	0.030	0.862
Follow-up period (months)	12.52 ± 5.06	13.49 ± 5.29	1.548	0.114
Minimal luminal diameter (mm)				
Baseline	1.93 ± 0.78	2.03 ± 0.80	1.049	0.295
Follow-up	1.46 ± 0.78	1.70 ± 0.80	2.590	0.010*
Δ	0.47 ± 0.58	0.33 ± 0.36	−2.628	0.009**
Diameter stenosis (%)				
Baseline	28.93 ± 23.14	27.14 ± 23.29	−0.649	0.517
Follow-up	46.59 ± 26.18	38.68 ± 25.06	−2.613	0.009**
Δ	17.66 ± 19.51	11.54 ± 12.12	−3.285	0.001**
Lesion location, *n* (%)				
LM	2 (1.6)	5 (3.0)	0.606	0.436
LAD	45 (36.0)	54 (32.5)	0.382	0.536
LCX	40 (38.1)	65 (39.2)	1.584	0.208
RCA	35 (28.0)	47 (28.3)	0.003	0.953
Revascularization, *n* (%)	21 (16.9)	14 (8.4)	4.834	0.028*
AMI, *n* (%)	5 (4.0)	2 (1.2)	2.373	0.123

MVD, multivessel disease; High RLP-C: ≥ 0.555 mmol/L; Low RLP-C: < 0.555 mmol/L; △, difference value; AMI, acute myocardial infarction.

* *P* value < 0.05.

** *P* value < 0.01.

**Table 4:** Correlation between RLP-C and other lipid parameters in well-controlled LDL-C individuals.

Variables	TG-FU	TG-BL	TC-FU	TC-BL	HDL-C-FU	LDL-C-FU	HDL-C-BL	LDL-C-BL
RLP-C-FU	.583**	0.074	.474**	.161**	−.247**	.052	−.086	.112
RLP-C-BL	.117*	.551**	.296**	.479**	.196**	.099	−.085	−.092

RLP-C, remnant lipoprotein particle cholesterol; TG, triglyceride; TC, total cholesterol; HDL-C, high density lipoprotein cholesterol; LDL-C, low density lipoprotein cholesterol; BL, baseline; FU, follow-up.

* *P* < 0.05.

** *P* < 0.01.

## Discussion

Patients with acute coronary syndrome have a high risk of major adverse cardiovascular events (MACE). Over a follow-up period of nearly 3 years, there was a progressive increase in the incidence of MACE with each additional metabolic risk factor, ranging from 7.8% (no risk factors) to 19.6% (five risk factors; HR: 1.18, 95% CI: 1.13–1.24 per metabolic risk factor) (10). The management of blood lipids is particularly important. High levels of LDL-C have a causal relationship with ASCVD, and reducing LDL-C can decrease the incidence of cardiovascular events (11). Nevertheless, the control of LDL-C remains unsatisfactory. An observational study conducted across 452 centers in 18 countries found that only 32.1% of patients with very high cardiovascular risk and 52.7% of patients with high cardiovascular risk achieved the LDL-C goals among the included cases (12). In this article, the achievement rate of LDL-C goals among post-PCI patients was only 37.8%. Health education, standardized medication usage, and follow-up management are prioritized in this endeavor.

However, even with LDL-C levels are controlled well, approximately 14.78% of patients occurred NTLs progression, in which case RLP-C appears to be particularly important. RLP-C may exert a potent atherogenic effect. Epidemiology shows that elevated RLP-C levels are significantly associated with ASCVD and are independent of other lipids in predicting adverse cardiovascular events. A recent study involving 17,532 American patients with coronary heart disease found that RLP-C correlated with myocardial infarction, coronary death, and stroke, even after controlling for LDL-C and apoB (4). Another study comprising 87,192 individuals of Copenhagen found that 22% of people with RLP-C ≥ 1 mmol/L were associated with a twofold increased risk of mortality from cardiovascular and other non-cancer causes (13).

In addition to clinical evidence and similar findings in genetics, this Mendelian randomized study once again confirmed the independent association of RLP-C and LDL-C on CAD, and also revealed that RLP-C may have a stronger effect on arteriosclerosis, The study additionally found that each 1 mmol/L rise in RLP-C and LDL-C was associated with OR values of 2.59 (95% CI: 1.99–3.36) and 1.37 (95% CI: 1.27–1.48) for CAD, respectively (3). In this article, after balancing other risk factors through propensity score matching, RLP-C remained an independent predictor for non-target lesion progression.

RLP-C is an important component of TRL metabolites. The comparison of the atherogenic potential between TRL and LDL involves several factors from a medical perspective. These include their plasma residence time, cholesterol burden, ability to penetrate and remain in the arterial intima, susceptibility to modification within the arterial walls, uptake rates by macrophages, and propensity to generate proinflammatory foam cells. These factors contribute to the observed higher risk of coronary heart disease associated with TRL compared to LDL alone (14). Additionally, similar conclusions have been reached by other studies (14–16).

Nevertheless, RLP-C is considered less significant than LDL-C in clinical practice currently. This may be due to the fact that the number of LDL particles is significantly higher than the others in general population, accounting for about 80%–95% of APOB particles, even in patients with hypertriglyceridemia, LDL particles remain a major driver of risk (17, 18).

Up to recently, RLP-C related studies mostly focus on cardiovascular adverse events and mortality as the primary endpoint events, but less on the progression of atherosclerotic lesions after PCI. There was an analysis of the correlation between RFP-C and In-Stent Restenosis (ISR) In 2019, it emphasized the importance of RLP-C in ISR, especially in diabetic patients (19). Nevertheless, various mechanisms contribute to the development, severity, and patterns of in-stent restenosis (ISR). These encompass biological or patient-specific factors, anatomical considerations, procedural variables, and characteristics of the stent, too many confounders weakened the effect of RLP-C in studies with small sample sizes (20). To avoid these effects, our study focuses on the progression of NTLs, so that the results can better reflect the progression of arteriosclerosis caused by metabolism. Interestingly, Our study revealed that individuals with heightened RLP-C levels exhibited a greater prevalence of diabetes, as discussed in the ISR study above. It is well known that the therapeutic targets of lipids for the prevention and treatment of ASCVD events in diabetic population are LDL-C and non-HDL-C, perhaps RLP-C can be used as a new therapeutic target, especially for people with low LDL-C and Hypertriglyceridemia.

RLP-C is closely associated with TG, and both are of significant prognostic value. The causal relationship between TG and CVD has been debated for years. Hypertriglyceridemia may reflect an increase in the number of certain triglyceride-rich lipoprotein particles that are associated with cardiovascular risk. Sometimes even large elevations of plasma triglycerides, such as those dominated by chylomicrons particles, which can cause acute inflammatory pancreatitis, do not enter the artery wall (21). This means TG may not directly contribute to plaque formation but may indirectly reflect residual particles and their cholesterol content, which play a role in the development of ASCVD sometimes. In this study, TG was higher in patients with NTLs progression and strongly correlated with RLP-C, but TG did not have a pathogenic effect similar to RLP-C in multivariate analysis. We tend to suggest that when triglyceride levels are high, estimates based on LDL-C levels alone are no longer sufficiently accurate to represent the lipid risk of atherosclerosis as TRL and cholesterol concentrations increase, especially in patients with obesity and diabetes who are highly associated with hypertriglyceridemia. During plaque formation, TG is broken down and do not accumulate within atherosclerotic plaque, Conversely, cholesterol carried by TRL is involved in the formation of dysregulated foam cells, suggesting that RLP-C may be more important than TG of atherosclerosis, as it is involved in pathological processes.

At present, the evidence of RLP-C causes arteriosclerosis is still limited and there is no therapeutic drugs targeting it. Our findings suggest that when LDL-C levels below 1.8 mmol/L, RLP-C is associated with an increased risk of lesion progression and revascularization, independent of other cardiovascular risk factors. The conclusion undoubtedly provides more evidence supporting that RLP-C causes arteriosclerosis.

## Deficiency

Firstly, our study was a retrospective analysis conducted at a single center. Second, most patients are admitted to the hospital for coronary angiography due to symptoms, rather than regular follow-up, resulting in a higher rate of lesion progression than the actual rate. Thirdly, in evaluating the progression of non-target lesions, there was no guidance from endovascular imaging. Both visual methods and QCA are prone to bias, making it challenging to accurately determine plaque burden and vulnerability.

## Conclusion

This study suggests that RLP-C could be a significant residual risk factor for coronary atherosclerosis progression. While LDL-C has garnered considerable attention in the current era of lipid reduction, it's essential to focus more on RLP-C to further mitigate ASCVD events. However, evidence is needed regarding the benefits of reducing RLP-C, and the development of more convenient and accurate measurement methods is also eagerly anticipated.

## Data Availability

The original contributions presented in the study are included in the article/Supplementary Material, further inquiries can be directed to the corresponding authors.
